# Effect of a Preventive Oral Health Program Starting during Pregnancy: A Case-Control Study Comparing Immigrant and Native Women and Their Children

**DOI:** 10.3390/ijerph18084096

**Published:** 2021-04-13

**Authors:** María García-Pola, Agueda González-Díaz, José Manuel García-Martín

**Affiliations:** Department of Surgery and Medical-Surgical Specialities, Faculty of Medicine and Health Sciences, University of Oviedo, 33006 Oviedo, Spain; aguedina@yahoo.es (A.G.-D.); garciamjose@uniovi.es (J.M.G.-M.)

**Keywords:** caries, primary prevention, immigrant, pregnant woman, health services, randomized trial

## Abstract

The objective was to evaluate whether including pregnant women in a preventive dental program prevented the appearance of caries in their children up to the age of 6, and whether the effect was similar in children of immigrant and non-immigrant women. In phase I, 90 pregnant women, 45 immigrants and 45 natives, were taught about the development and prevention of caries. In phase II the oral health of their children at the age of 6 (*n* = 90) was evaluated, along with a control group of children of natives and immigrants of the same age (*n* = 90). A survey was used to determine participants’ backgrounds and habits. A multivariate study of the results was performed using R-core software. The number of children without caries was 128 (71.1%), whereas 52 (28.9%) had caries, 15 from the protocol (16.67%) and 37 from the control group (41.11%), with statistically significant differences (*p* < 0.001). The mean number of caries for the children in the protocol was 0.62 ± 2 and in the control group it was 1.88 ± 2.9 (*p* = 0.001). In the multivariate analysis the risk of developing caries was higher for the condition of being the child of an immigrant (OR = 11.137), inadequate oral health (OR = 4.993), the children being overweight at the age of 6 (OR = 10.680), and the consumption of candies (OR = 5.042). In conclusion, the preventive protocols started during pregnancy reduced caries in participants’ children, which suggests that these protocols should be encouraged. Because immigrant children are more vulnerable to caries, they and their parents should be included in preventive programs once they arrive in the host country.

## 1. Introduction

Caries in the primary teeth of preschool children is commonly referred to as early childhood caries (ECC) [1]. ECC is defined by the presence of one or more caries lesions, cavitated or non-cavitated, absent teeth due to caries, or filled part of the primary tooth surface in a child ≤71 months old [2,3]. ECC is a multifactorial disease, and its prevalence varies between 1% and 12% in developed countries [4], but can reach up to 90% in children [5].

It is accepted that oral health is affected by personal, family, and socio-community factors [6], but the possible transmission of factors to a child determined by the mother’s health must also be considered [7]. Other factors have been proposed such as the mode of delivery, low birth weight and prematurity [8].

Risk factors based on children’s habits include consumption of sugar-sweetened beverages (SSB) [9,10], sweets [11], or snacks [12] between or during meals. Another aspect, about which there is less consensus, is being overweight or obese; some studies have shown a connection between children’s obesity and the presence of caries while others have not, and one systematic review has even shown the opposite effect [13].

Socio-economic factors are no less important [6]. Children who live in precarious economic conditions and immigrants have been shown to have a poorer standard of oral health [14], hence the American Academy of Pediatric Dentistry still consider these to be high and moderate risk factors for caries [15].

It is important to reduce caries because of the pain it causes, as well as masticatory difficulty [16], which in some cases can lead to psychomotor disorders [17] and reduced quality of life [18]. Early childhood caries can also lead to problems with learning development due to school absenteeism [19]. Childhood caries has also been connected to caries in adulthood, which is another reason to prevent it at early ages [20,21].

Preventive studies aimed at early childhood caries, begun while the mother is pregnant, have been able to reduce caries. The effects of preventive dental programs (PDP) can vary depending on factors such as the age the child is examined, with rates of children who are free from caries fluctuating between 35% and 97% [22,23].

Between 16% and 83% of pregnant women use dental health services [24]. Both native and immigrant pregnant women have been provided with voluntary programs by the public oral health system that focus on prevention and diagnosis of oral diseases, as well as instruction in healthy habits for prevention of oral pathologies [25].

One of the universal objectives for the year 2020 was to increase the number of 6-year-old children who were free of caries, and to understand the risk factors for caries in the population under examination in order to establish preventive measures based on their needs [26]. The hypothesis of this study was that providing pregnant women with preventive dental care and adequate information about oral health habits and healthy nutritional practices would prevent their children from suffering from caries up to the age of 6. The objective of the study was to determine and compare the effects of the preventive dental program for native and immigrant women beginning during pregnancy and analyzing their children at the age of 6 years old.

## 2. Materials and Methods

### 2.1. Study Design

The present prospective case-control study was carried out in two phases following the Strengthening the Reporting of Observational Studies in Epidemiology (STROBE) criteria [27]. Phase I was in instruction of prevention and oral health habits in pregnant patients who attended a Public Health Center. Phase II was the evaluation of the preventive effect on the expression of caries in participants’ children at the age of 6, comparing that with the presence of caries in other 6-year-old children whose mothers did not attend the preventive dental program (PDP).

### 2.2. Participants and Setting

The population in the study attended the Oral Health Unit (OHU) at Vallobin-La Florida Health-care Center and Special Health Care Area Teverga-Proaza-Quiros Area, which are in Health Sector IV in Oviedo and part of the Health Service of the Principality of Asturias. The study was carried out at a dental clinic in the center between 2010 (phase I) and 2019 (phase II).

Pregnant patients were recruited consecutively [28,29], creating the prevention group for Phase I of the study. Participants were added to the study until the necessary representative sample size was reached, with immigrants and natives equally represented.

When the children were 6 years old, the mothers from the preventive group were contacted by letter by the OHU, as were the cohort of 6-year-old children whose mothers had not been in the program. The former was the prevention study group, the latter was the control group for phase II. For the children in the control group, the data from 45 native children and 45 immigrant children were collected consecutively. All of the children in the control group were 6 years old during the same year as the children of study group.

To be classified as immigrants, the pregnant mothers in phase I and the children in phase II must have been recorded as foreigners in the Healthcare database [30] and have been living in Spain for less than 3 years (at the time of phase I for the prevention group, and the time of phase II for the control group).

The inclusion criteria for the pregnant women to take part in the PDP were as follows [23]: (1) ≥18 years old, healthy women, without the need for topical or systemic treatment; (2) consent to take part in the study; (3) attend the informative talk about prevention; (4) consent to and undergo the oral exploration; and (5) fill in the survey.

The inclusion criteria for the 6-year-old cohort in phase II were as follows [23,28,31]: (1) consent for them to participate in the study from legal guardians; (2) belong to the 6-year-old age cohort, either children from the pregnant women who took part in the preventive study or children whose mothers did not belong to the prevention group; (3) consent to the exploration; and (4) having the survey completed voluntarily by legal guardians.

The exclusion criteria were: (1) not consenting to be involved in the study; (2) refusal by pregnant women or legal guardians to complete the survey; (3) lacking some socio-demographic or survey data; and, (4) in the immigrant group, living in Spain continuously for more than 3 years (at the relevant timepoint).

### 2.3. Data Sources

The dependent variable was the experience of caries in all groups as an indicator of oral health.

A protocol was developed based on dental health criteria that in our OHU are considered. We used an Oral Health Care Guide for pregnant women and children, which included caries risk factors [32]. Thus, we have considered the following independent variables for the pregnant patients: demographics (age, continent of origin), harmful habits (alcohol and tobacco), and oral fluoride intake. Variables related to food habits were grouped into 3 questions about the consumption of snacks, sweets, and sugar-sweetened beverages (SSB), which asked about how often they were consumed in the three months before the intervention. We also recorded the DMFT (decayed, missing, filled permanent teeth) index and the O’Leary Index as an indicator of oral hygiene [33].

The variables for the 6-year-old children, collected through 16 questions, were: demographics (gender and continent of origin) and variables collected in the survey about hygiene, food habits, and the number of dental visits. Another variable we recorded was birth weight, defined as: overweight (>4000 g), normal weight (between 2500 and 4000 g), and low weight (<2500 g) [34]. The type of lactation during the first six months was divided into 3 categories: maternal lactation, artificial, and mixed or complementary. The Body Mass Index (BMI) of the child was classified in three categories, according to WHO Child Growth Standards for boys and girls from 5 to 10 years old, as normal weight, overweight (percentile 85), or obese (percentile 95) [35].

Variables related to the children’s backgrounds were obtained through their medical history or provided by their guardians.

### 2.4. Program Description

In phase I, the pregnant group attended a 60-min talk given in person by the dentist, informing them about oral hygiene (tools and how to use them, toothbrush, dental floss, toothpaste, mouthwash, and the benefits of xylitol) and healthy eating habits for the mother (a list of foods that would be detrimental to their general and oral health or not) and for the baby (the use of fingerstalls, pacifiers, bottles, and food habits). They were also informed about how caries occurs and how dental plaque is involved in its development. The oral examination was carried out in the third and sixth months of pregnancy, and after delivery. In addition, a professional prophylaxis was carried out in the sixth month of pregnancy, followed by the application of acidulated phosphate fluoride dental gel in 1.23% (for 1 min) [36]. The values for the pregnant woman in this study are from the first oral examination.

In phase II, before the children’s exploration, their guardians were given a survey form to collect information. The clinical examination was performed by a single researcher (G-M), who instructed and advised about the procedures, the purpose of the study, and how to fill out the survey, along with helping with any language difficulties.

Caries diagnosis followed the WHO criteria for community programs and were expressed for pregnant women using the DMFT index, and the DMFT index for children (decayed and filled temporary teeth) [37]. Examination was carried out under artificial light in a dental chair, using dental mirror, periodontal and gauze. To measure the level of oral hygiene we used the O’Leary Index via erythrosine staining, which is given by the number of stained surfaces x 100/the total number of surfaces present. Good hygiene would be indicated by 0–15% positive staining, less good hygiene by staining of 16–49%, and poor hygiene by staining over 50% [33].

The study was approved by the Regional Clinical Research Ethics Committee of the Principality of Asturias (73/10). All legal guardians were informed of the study objectives before being asked to sign their final consent.

### 2.5. Study Size

The sample size was calculated applying a 10% margin of error, confidence levels of 95%, and a proportion of expected response of 50% of participants [38]. Based on data provided by the National Statistical Institute (Instituto Nacional de Estadística de España) [39], the proportion of children with immigrant status was estimated to be 10% per cohort. The Oral Health Unit at the Health Center sees approximately 225 6-year-olds per year, giving an estimate of 900 children in the specific population during the study period, and thus four years of recruitment.

**Figure 1:**
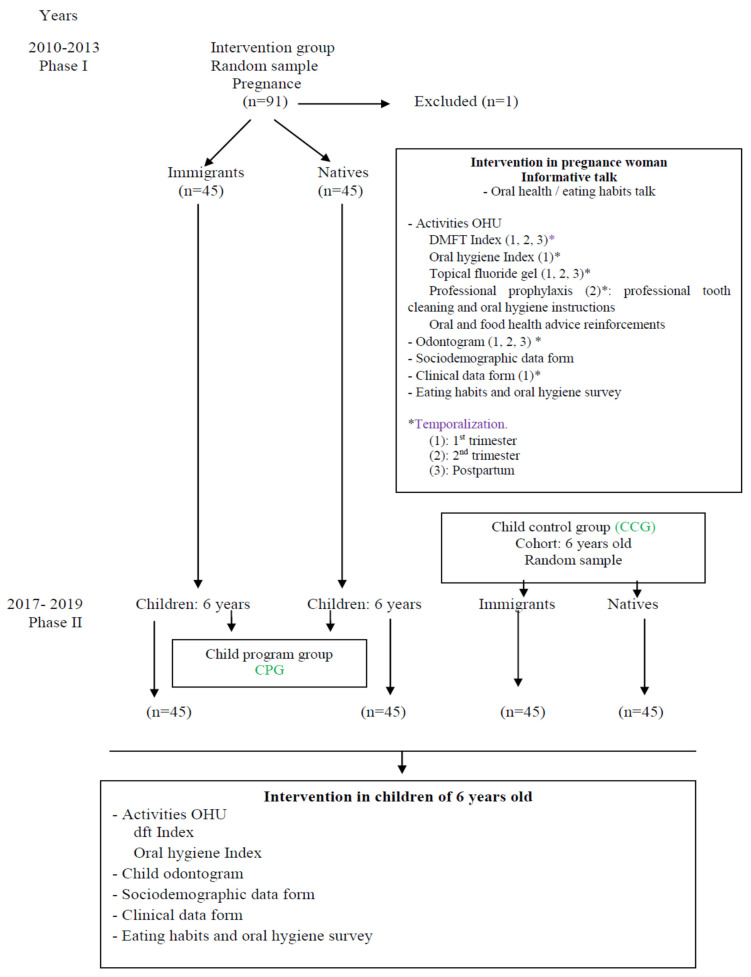
Study design. Flow diagram of the participants. DMFT: decayed, missing, filled teeth. dft: decayed, filled teeth. OHU: Oral Health Unit. CPG: Child program group. CCG: Child control group.

Once the representative sample size of immigrant children was established, in order to carry out the study, half of the children would be from the women attending the prevention program group (CPG) and the other half would make up the control group (CCG). After one participant was excluded for not having filled in the survey properly, the sample was reduced to 90 women, 45 immigrants (IP) and 45 natives (NP). The selection process is given in Figure 1.

### 2.6. Statistical Methods

The data were collected in a database and processed in the Statistical Consulting Unit of the Univerty of Oviedo, using R (R Development Core Team, version 3.6, 2018). First, we performed a descriptive analysis of each variable. Then we split the following variables into dichotomous categories: mother’s age (≤35 years old) [40]; DMFT (0, ≥1); level of oral hygiene (good, if ≤15%; and inadequate, if >16%), the number of dental visits before the study (0, ≥1), birth weight (normal, low weight), weight at 6 years old (normal, vs. overweight), and the consumption of sweets, SSB, and snacks (never vs. some times a week). To compare the measures in the quantitative variables we used the Student t test, and the relationships between the qualitative variables were examined using Pearson’s chi-squared test or Fisher’s test to verify the hypothesis about the expected frequencies, with the *p* value being statistically significant if it was ≤0.05. We used the Fisher test for small/undersized variables. We then used logistic regression models to measure the association of caries in children, and estimated the odds ratio (OR), adjusted to the selected covariates, if in the bivariate analysis the *p* value was ≤0.2.

## 3. Results

### 3.1. Characteristics of Pregnant Women Included in the Program. Phase I

Table 1 gives the characteristics of the participating pregnant women, their continents of origin, and the details of their habits. There were no significant differences in the numbers of immigrant (IP) and native (NP) women who were over 35 years old (*p* = 0.371).

The mean DMFT Index in the IP group was 8.33 (±6.66) and in the NP group it was 8.07 (±6.058), ranging between 0 and 22 in the two groups. There were no statistically significant differences between the two groups in terms of the presence of caries (*p* = 0.245) or the mean number of caries (*p* = 0.843).

An O´Leary Index ≤15% was recorded in 35.6% of the IP group (*n* = 16) and in 55.6% of the NP group (*n* = 25) with no statistically significant differences (*p* = 0.097).

### 3.2. Children’s Sociodemographic Characteristics. Phase II

Table 2 shows the differences in habits between children from the study group (children in the preventive group, CPG) and control group (children in the control group, CCG).

The origins of the immigrant children in the CG (ICCG) in phase II was as follows: South America (*n* = 31; 68.9%); Africa (*n* = 6; 13.3%); Europe (*n* = 6; 13.3%), and Asia (*n* = 2; 4.4%). There were no significant differences between the origins of the IP group in phase I and the immigrant children in the CG (ICCG) in phase II (*p* = 0.357).

The CPG exhibited better hygiene (*p* = 0.005) and used fluoride toothpaste more (*p* = <0.001), while the CCG used fluoride mouthwash more (*p* < 0.001). In addition, the CCG ate sweets more often (*p* = 0.023), and more of them were overweight at the age of 6 (*p* = 0.005).

### 3.3. Analysis of the Presence of Caries

The experience of caries in each group of children is shown in Table 2. The number of caries ranged from 0 to 13 in the CPG and from 0 to 12 in the CCG, with the highest numbers in both cases being in immigrant children. Over a quarter of the children (52; 28.9%) had caries (28.9%), 15 from the CPG (16.67%) and 37 from the CCG (41.11%), with the difference being statistically significant (*p* < 0.001).

The mean number of caries in the CPG was 0.62 ± 2, while in the CCG it was 1.88 ± 2.98, with statistically significant differences (*p* = 0.001). Comparing the mean number of caries between the immigrant subgroups, ICPG (1.07 ± 2.66) and ICCG (3.51 ± 3.48), there were also statistically significant differences (*p* < 0.001), however, there were no differences in the natives (NCPG, 0.18 ± 0.78, vs. NCCG, 0.24 ± 0.61; *p* = 0.652).

The presence of caries in the CPG was statistically significantly inversely associated with the use of fluoride toothpaste (*p* = 0.026) and directly associated with the consumption of sweets (*p* = 0.023). In the ICPG, the relationship with caries was non-maternal lactation (*p* = 0.035) and also the consumption of sweets (*p* = 0.014).

The presence of caries in the CCG was statistically significantly associated with boys (*p* = 0.015), pre-term pregnancy (*p* = 0.013), cesarean delivery (*p* = 0.013), non-maternal lactation (*p* = 0.018), low birth weight (0.045), being overweight at the age of 6 (*p* < 0.001), the number of dental visits (*p* = 0.003), inadequate oral hygiene (*p* < 0.001), the consumption of snacks (*p* = 0.037), and the consumption of sweets (*p* = 0.003).

Table 3 gives the results of the logistic regression analysis of the pregnant women. There was a higher risk of caries in natives who smoked (OR = 3.990). There was also a higher risk of caries for both immigrants and natives in the presence of insufficient oral hygiene (*p* = 0.004; OR = 22.286) and the consumption of sweets (*p* = 0.039; OR = 11.137), with the effect increasing in the multivariate analysis, OR = 40.289 and OR = 22.286, respectively.

In the logistic regression analysis (Table 4), the children from the CG exhibited a higher risk of caries than the children in the CPG (OR = 3.491), and there was a greater risk of caries for immigrants (OR = 6.009). The habits that increased the risk of caries were inadequate oral health (OR = 8.826), not using fluoride toothpaste (OR = 2.654), eating sweets (OR = 3.789), and being overweight at the age of 6 (OR = 7.000).

In the multivariate analysis, being an immigrant mother-to-be increased the risk factor of caries in children (*p* = 0.007; OR = 12.661). The multivariate analysis also confirmed that the risk of caries increased in the condition of being an immigrant (OR = 11.898), inadequate oral hygiene (OR = 4.993), the consumption of sweets (OR = 5.042), and the children being overweight at the age of 6 (OR = 10.680). However, not attending the protocol was not associated with a greater risk of caries.

## 4. Discussion

The aim of this prospective study was to evaluate how effective a preventive dental program (PDP), started during pregnancy, was in preventing ECC. We also aimed to compare any differences in the effect of the PDP between the 6-year-old children of immigrant and non-immigrant mothers. The PDP had a positive effect, reducing the prevalence of caries in 6-year-old children, and increasing the knowledge of caries indicators in the study population. None of the children dropped out of the study, reinforcing its effect.

The native and immigrant mothers-to-be who took part in the program were a relatively homogeneous group in terms of age and in terms of DMFT status (88.9% in immigrants vs. 80% in natives). The multivariate analysis showed that the condition of being an immigrant did not increase the risk of caries compared to native mothers-to-be. These data are close to the prevalence rates of 92.1%, seen in a socially deprived population such as Argentina [31], and very different to the data from other countries, such as Germany (14%) [41] and the USA (28.3%) [42].

The design of the PDP in our study was similar to programs in previous studies [23,41,43,44,45,46,47,48]. They included hygiene recommendations, advice about diet, and healthy habits in the prevention of caries in pregnant women and their children. Some previous protocols also included dental cleaning by a professional and a fluoride treatment [23,41,43]. Subsequent check-ups were at varying ages of the children. The most notable difference between those studies, and evident in the results, was when the evaluation was performed. The exploration for the presence of caries was at 24 months old [45,47], 30 months [22,48], 3.5 years [23], 4 years [41], and well into adolescence, at 13, and 18 years old [44,46].

Fewer studies of this type of PDP have done evaluations at the age of 6. In the study by Gomez et al., in a Chilean population, 89% of children were found to be free from caries compared to 50% in the control group [23]. In the study by Plutzer et al. [29], in Australia, 67% of the children in the intervention group were free of caries, compared to 58% in the control group. That study only described the statistically significant differences in the mean number of caries. These numbers differ from our study in which the immigrant children in the control group had worse levels of caries than the cited control groups.

In our study we included possible confounding maternal and child-related factors which may influence caries. This is where a multivariate analysis is needed. On analyzing maternal influencing factors in the development of caries in their children, the multivariate analysis could only confirm an association with the condition of being an immigrant mother, (OR = 12.661). Association with other factors described in previous studies, such as the age of the mother [49], smoking habits [50], the activity of caries [51], or the consumption of sweets or snacks [50], were not confirmed.

More than a hundred factors related to ECC have been described [52] and the connection between ECC in the control group and some of those factors can be seen in our study. However, from the multivariate analysis in our study, which included 16 child-related factors and 8 maternal factors, we deduce that the four co-pathogenic pillars of ECC in the study sample were: the condition of being an immigrant (in the mother and also the child); inadequate hygiene; the consumption of sweets; and being overweight at the age of 6. Other variables that had an effect, such as the number of dental visits, were more difficult to support, as in the PG the immigrant children had more visits, while in the CCG it was the native children who went to the dentist more. Nevertheless, a recent publication has shown that immigrant children, compared to those born in the United States, visit the dentist less once a year preventively and are less likely to be able to access dental care [53]. Similarly, it was difficult to support the predominant trend of caries in boys noted in the literature [52].

The prevalence of ECC in 6-year-old Spanish children in 2005/06 was 31.3% [54], and in immigrant children it was 47.23% [55], while more recently, in low-income areas it reached 77.3% [56], and 83% [57]. The proposed expectations for the year 2020 in Spain, collected in the prospective Delphi study, were to keep 75% of 6-year-old children free from caries and with a DMFT of 2.4 [58]. Based on this proposal, the program achieved its objectives in spite of considering the condition of being an immigrant a risk factor. Three-quarters (75.6%) of the children born from immigrant mothers were free of caries, and the average DMFT was 1.07. We achieved better results in our study than Harrison et al. [22], who observed indigenous children at the age of 30 months in a motivational program, 35% of children in their study group and 25% of the children in their control group were free of caries.

Inadequate hygiene was another risk factor for ECC (OR = 4.993), more evidently found between immigrant children in the control group. This risk indicator confirms the need for good hygiene to prevent ECC, a proposal suggested in the last European consensus Delphi [59], which our study verified as something that can be achieved through maternal instruction and learning.

Although the literature suggests that ECC is also associated with the consumption of SSB [9], and chips [12], in our sample, despite ECC in the control group being significantly associated with the consumption of chips (*p* = 0.037), only the consumption of sweets manifested as a risk factor (OR = 5.042). On examining this habit, we found that the children in the control group tended to eat more sweets, immigrant children even more so, and they also were more likely to be overweight at the age of 6. The consumption of sweets, cookies, and chocolates an average of 9 times per week is associated with cultural factors such as being an immigrant [60]. The condition of being an immigrant [61], race, and ethnicity [62], are also correlated to obesity.

There have been attempts to associate a higher prevalence of caries in children who are overweight or obese [63,64], describing primary teeth as more sensitive [13]. Other studies have suggested that this may only affect permanent teeth [65], and yet more studies have indicated that caries may be more prevalent in underweight and overweight children [66]. The risks of caries have also been seen to begin from very young ages, and increase from 2-year-old children upwards. Prikaneron et al., described 1.92 to 3.6 times higher prevalence of caries in 6-year-old children who were overweight or obese (respectively) [67]. A similar situation was described by Hong et al., who found a statistically significant connection between caries rates and obesity in children aged between 60 and 72 months, while they found no association in younger children [68]. Interpretations of these different results must be made cautiously because the international measure is adapted to percentiles. Children’s BMIs constantly change as they grow [69], which is why the age intervals should be narrowed down to establish whether there is an association with being overweight or obese. Data analysis with wide age ranges make comparisons difficult which may be another reason why our results agree [70], or disagree, with results from other European studies [71].

One of the limitations of our study is the time needed to obtain a sample from immigrant mothers-to-be compared to non-immigrants. Secondly, part of our study was a survey, and the data related to the habits should be interpreted in the light of that. However, the study’s strengths included exploring the presence of caries using tools recognized by the WHO, and the effectiveness of oral hygiene via the plaque index, as well as having a representative sample of the population where the study was carried out.

## 5. Conclusions

In conclusion, our study confirmed our hypothesis, that women, immigrant and not immigrant, who participate in a dental prevention protocol reduce the risk of caries in their children at the age of 6, indicating that these programs should be encouraged and maintained. Immigrant children are the most vulnerable, and health authorities should reinforce public health intervention not only to include dental preventive programs for pregnant immigrant women, but also for immigrant children, who should both be included in caries prevention programs once they arrive in the host country.

We confirm that the three authors have contributed accordingly to the design of study, writing and editing of this research. García-Martín has contributed substantially to exploring the patients. All authors approve this version to be published and agree to be accountable for this work.

## Figures and Tables

**Table 1:** Demographic characteristics, oral health and habits of the group of pregnant women included in the oral health preventive program (phase I)**.** DMFT: decayed, missing, filled teeth. In bold letters, statistically significant *p*-values.

	Immigrants	Natives	*p* Value
Variables	45 (50%)	45 (50%)	
Continent			
South America	29 (64.4%)		
Africa	7 (15.6%)		
Europe	7 (15.6%)		
Asia	2 (4.4%)		
Age			
Mean	30.29 (±6.178)	34.42 (±4.639)	**0.001**
Range (years)	18–40	19–41	
≤35 vs. >35	32/13	28/17	0.843
DMFT			
Mean	8.33 ± 6.66	8.07 ± 6.05	0.843
Decayed (yes)	40 (88.9%)	36(80%)	0.245
Range	0–22	0–22	
O´Leary Index			0.097
≤15%	16 (35.6%)	25 (55.5%)	
16–49%	21 (46.7%)	17 (37.8%)	
≥50%	8 (17.7%)	3 (6.7%)	
≤15% vs. >16%			0.057
Tobacco user (yes)	0	5 (11.1%)	0.056
Alcohol drinkers (yes)	0	1 (2.2%)	1
Use oral fluoride (yes)	0		0
Sugar-sweetened beverage (yes)	28 (62.2%)	26 (57.8%)	0.667
Consumption of snacks (yes)	1 (2.2%)	8 (17.8%)	**0.03**
Consumption sweets (yes)	18 (40.0%)	14 (31.1%)	0.378

**Table 2:** Characteristics of the children’s variables and their relationship between the different groups: Children from oral health preventive program, children from control group. SBB sugar-sweetened beverages. NA: not applicable. In bold letters, statistically significant *p*-values. CPG: child program group. CCG: child control group.

	Children Program Group		Children Program Group		Children Control Group	
	vs. children Control Group		Immigrant Native		Immigrant Native	
Variable	(*n* CPP/*n* CCG)	*p* Value	*n* (%) *n* (%)	*p* Value	*n* (%) *n* (%)	*p* Value
*Experience of caries*						
Caries (yes)	15/37	**<0.001**	11 (24.4) 4 (8.8)	**0.048**	30 (66.6) 7 (15.5)	**<0.001**
Mean	0.62 ± 2/1.88 ± 2.9	**0.001**	1.07 ± 2.6 0.18 ± 0.7	**0.036**	3.5 ± 3.4 0.24 ± 0.6	**0.001**
Range	(0–13)/(0–12)		(0–13) (0–5)		(0–12) (0–2)	
*Gender*		0.881				
Girl	42/43		21 (46.7) 21 (46.7)		21 (46.7) 22 (48.9)	
Boy	48/47		24 (53.3) 24 (53.3)		24 (53.3) 23 (51.1)	
*Full term pregnancy (yes)*	85/78	0.074	41 (91.1) 44 (97.7)	0.361	42 (93.3) 36 (80.0)	0.063
*Vaginal delivery (yes)*	73/74	0.847	38 (84.4) 35 (77.7)	0.419	41 (91.1) 33 (73.3)	**0.027**
*Weight at birth (normal)*	81/79	0.635	43 (95.5) 38 (84.4)	0.157	38 (84.4) 41 (91.1)	0.334
*Lactation (maternal)*	48/55	0.292	29 (64.4) 19 (42.2)	**0.035**	31 (68.8) 24 (53.3)	0.13
*Mixed*			9 (20.0) 7 (15.6)		7 (15.6) 5 (11.1)	
*Artificial*			7 (15.6) 19 (42.2)		7 (15.6) 16 (35.5)	
*Weight at six year (normal)*	84/69	**0.005**	40 (88.9) 44 (97.8)	0.203	31 (68.9) 38 (84.4)	0.081
*Overweight*			4 (8.8) 1 (2.2)		13 (28.9) 3 (6.6)	
*Artificial*			1 (2.2) 0 ()		1 (2.2) 4 (8.8)	
*Visits at dentist (1/>2)*	53/44	0.897	8/37 45/0	**<0.001**	31/14 23/22)	0.085
*O´Leary (≤15%* *)*	74/61	**0.025**	34 (75.6) 40 (88.9)	0.098	24 (53.3) 37 (82.2)	**0.01**
*16–49%*			7 (15.6) 2 (4.4)		13 (28.9) 6 (13.3)	
≥50%			4 (8.9) 3 (6.7)		8 (17.8) 2 (4.4)	
*Use of dentifrice free fluoride (yes)*	2/14	**0.004**	1 (2.2) 1 (2.2)	1	2 (4.4) 12 (25.7)	NA
*Never*			44 (97.8) 44 (97.8)		43 (95.5) 33 (73.3)	**0.002**
*Sometime/week*			0 (0) 1 (2.2)		1 (2.2) 0 (0)	
*Sometime*			1 (2.2) 0 (0)		1 (2.2) 12 (26.6)	
*Use of fluoride dentifrice (yes)*	88/67	**<0.001**	43 (95.6) 45 (100)	1	36 (80.0) 31 (68.8)	0.227
*Never*			2 (4.4) 0 (0)		9 (20.0) 14 (31.1)	**0.002**
*Sometime/week*			8 (17.8) 23 (51.5)		13 (28.9) 29 (64.4)	
*Sometime*			35 (77.8) 22 (48.9)		23 (51.1) 2 (4.4)	
*Use of mouth rinse (yes)*	0/4	0.121	0 (0) 0 (0)	1	4 (8.9) 0 (0)	0.117
*Never*					41 (91.1) 45 (100)	
*Sometime*					4 (8.89) 0 (0)	
*Use of fluoride mouth rinse (yes)*	1/16	**<0.001**	0 (0) 1 (2.2)	1	6 (13.3) 10 (22.2)	0.27
*Never*					39 (86.6) 35 (77.8)	
*Sometime/week*			0 (0) 1 (2.2)		6 (13.3) 5 (11.1)	
*Sometime*					0 (0) 5 (11.1)	
*Consumption of SSB (yes)*	0/0	NA	0/0	NA	0 (0)	NA
*Consumption of chips (Sometime a week/never)*	22/28	0.318	0 (0) 22 (48.9)	**<0.001**	4 (8.9) 24 (53.3)	**<0.001**
*Consumption of sweets (Sometime a week/never)*	31/39	**0.003**	8 (17.8) 23 (51.1)	**0.001**	27 (60.0) 12 (26.7)	**0.001**

**Table 3:** Caries risk in pregnant women. Univariate and multivariate logistic regression. OR: odd ratio. CI: confidence interval. In bold letter, statistically significant p-values and valid OR.

	Univariate Analysis			Multivariate Analysis		
Variable (Reference in Parentheses)	*p* Value	OR Unadjusted	(95% CI)	*p* Value	OR Adjusted	(95% CI)
Native (Immigrant)	0.251	2.000	(0.630–7.027)	0.881	0.861	(0.112–6.380)
Age (>35)	0.311	2.020	(0.572–9.49)	0.723	1.382	(0.230–9.261)
Hygiene (Insufficient)	**0.004**	**22.286**	(4.108–415.678)	**0.003**	**40.289**	(5.499–958.441)
Alcohol Use (Yes)	NA					
Tobacco Use (Natives)	**0.046**	**3.990**	(1.133–18.755)			
Use of dentifrice free fluoride (No)	0.347	1.737	(0.552–5.743)	0.357	2.900	(0.322–34.554)
Use of fluoride dentifrice (No)	0.689	0.791	(0.240–2.489)	0.649	1.720	(0.171–21.486)
Fluoride mouth rinse (No)	0.512	1.612	(0.327–6.220)	0.925	0.912	(0.121–6.106)
Fluoride mouth free rinse (No)	0.613	1.364	(0.383–4.424)	0.961	0.958	(0.162–5.340)
Comsumption of SSB (Sometime a week)	0.134	0.355	(0.076–1.247)	0.072	0.216	(0.034–1.041)
Comsumption of chips (Sometime a week)	0.700	0.654	(0.034–4.025)	0.601	0.457	(0.012–6.932)
Consumption of sweets (Sometime a week)	0.039	**8.956**	(1.653–166.848)	**0.041**	**11.137**	(1.546–238.026)

**Table 4:** Caries risk of immigrant and native children according to the variables considered for the mothers and the child’s. Univariate and multivariate analysis. NA: not applicable. OR: odd ratio. CI: confidence interval. In bold letter, statistically significant *p*-values and valid OR.

	Univariate Analysis			Multivariate Analysis		
	*p* Value	OR Unadjusted	(95% CI)	*p* Value	OR Adjusted	(95% CI)
*Mothers variables*						
Immigrant	**0.046**	**3.990**	(1.133–18.755)	**0.007**	**12.661**	(2.362–102.707)
Age (>35)	1		0.318–3.497			
Caries (Yes)	0.318	2.935	(0.516–55.509)			
Tobacco Use (Yes)	0.375	3.083	(0.135–32.026)			
Alcohol Use (Yes)	NA					
Hygiene (Insufficient)	**0.039**	**4.108**	(1.190–19.106)	0.145	**3.168**	(0.741–18.169)
Use of dentifrice free fluoride/never	0.117	2.678	(0.833–10.353)			
Use of fluoride dentifrice/never	0.116	0.293	(0.113–1.218)			
No Fluoride moth rinse/never	0.604	0.687	(0.181–3.365)			
Fluoride mouth rinse/never	0.36	1.882	(0.537–8.820)			
Consumption of sweets (sometimes/week)	**0.035**	0.295	(0.089–0.912)	**0.045**	0.244	(0.056–0.936)
Consumption of chips (sometimes/week)	0.73	1.430	(0.151–10.302)			
Consumption of SSB (sometimes/week)	0.605	0.704	(0.182–2.728)			
*Children variables*						
Immigrant	**<0.001**	**6.009**	(2.908–13.309)	**<0.001**	**11.898**	(3.244–54.480)
No preventive program	**<0.001**	**3.491**	(1.770–7.164)	0.514	1.479	(0.437–4.719)
Sex (boy)	0.243	1.476	(0.772–2.865)	**0.039**	**2.989**	(1.100–9.025)
Hygiene (deficient)	<0.001	**8.826**	(4.207–19.255)	**0.003**	**4.993**	(1.763–14.848)
Visits at dentist (1)	0.173	1.601	(0.823–3.203)	**0.039**	**3.170**	(1.091–9.982)
Full term pregnant	0.057	0.137	(0.008–0.701)	**0.025**	0.024	(<0.001–0.381)
Lacta (no maternal)	0.282	0.695	(0.354–1.339)			
Delivery (caesarean)	0.798	0.859	(0.251–2.678)			
Weight at birth (low weight)	0.249	1.758	(0.650–4.543)			
Weight at 6 years (overweight)	**<0.001**	**7.000**	(2.954–17.686)	**0.018**	**10.680**	(1.823–102.488)
Use of dentifrice free fluoride (never)	0.429	0.650	(0.228–2.004)			
Use of fluoride dentifrice (never)	**0.027**	**2.654**	(1.109–6.330)	0.638	1.746	(0.187–19.572)
No fluoride moth rinse (never)	0.362	0.397	(0.047–3.380)			
Fluoride mouth rinse (never)	0.542	0.721	(0.258–2.197)			
Cosumption of SSB	NA					
Cosumption of chips (sometime a week)	**0.049**	0.444	(0.188–0.962)	0.416	0.561	(0.132–2.257)
Consumption of sweets (sometime a week)	**<0.001**	**3.789**	(1.947–7.547)	0.002	**5.042**	(1.882–14.591)

## Data Availability

Data is contained within the article.

## Abbreviations

OHPP (oral health preventive program); IP (immigrant pregnant woman); NP (native pregnant woman); IC (immigrant children); NC (native children); CPG (children from preventive program group); CCG (children from control group); ICPP (immigrant children from preventive program); NCPP (native children from preventive program); ICCG (immigrant children from control group); NCCG (native children from control group), PDP (preventive dental program).
